# Encouraging Cross-Disciplinary Collaboration and Innovation in Epidemiology in Japan

**DOI:** 10.3389/fpubh.2021.641882

**Published:** 2021-03-31

**Authors:** Hiroshi Yokomichi, Mie Mochizuki, Zentaro Yamagata

**Affiliations:** ^1^Department of Health Sciences, University of Yamanashi, Yamanashi, Japan; ^2^Department of Pediatrics, University of Yamanashi, Yamanashi, Japan

**Keywords:** epidemiology, variation, diversity, public officer, clinician-researcher, job change, collaboration, statistical indicator

## Abstract

**Background:** Scientific innovation is often achieved through the intersection of ideas from different fields. However, barriers prevent non-epidemiologists from cultivating interests in epidemiology or undertaking epidemiologic work. In this study, we evaluated changes in the diversity of research topics in an epidemiologic journal over time. We aimed to understand how epidemiologists and non-epidemiologists communicate about epidemiologic data and how this impacts innovation in the field.

**Methods:** We categorized the topics of articles published in the *Journal of Epidemiology* during the early and late 2010s based on their titles. We calculated the Shannon–Weaver diversity index (H′) to measure changes in the diversity of topics addressed by published articles.

**Results:** Comparing 2011–2013 with 2017–2019, there was no significant change in the diversity of article topics (H′ = 4.25 and 4.21, respectively) published in the *Journal of Epidemiology*.

**Conclusion:** To encourage healthcare providers and public administrators to conduct or comment on epidemiologic studies, epidemiologists should present their findings in easily understood language with appropriate and relevant statistical indicators and useful illustrations. Bringing experience from other specialties into epidemiology may yield new findings from epidemiologic data because of the exposure of non-epidemiologists to different values, workplaces, and occupations. Collaboration among professionals from varied backgrounds and with varied occupational experiences may help to promote scientific innovation by broadening perspectives. In addition, a range of professional experiences may enable individuals to solve difficult research questions more easily by themselves.

## Introduction

Innovation in science is often achieved when new ideas and technologies come together (1). In epidemiology, this may occur when people from diverse backgrounds read or are involved in a study (2). Research results should be transparent to everyone (3). Research papers, particularly epidemiologic papers with public value (4), should be written to ensure that they can be broadly understood.

In Japan, there are many societal expectations regarding professional roles (5). The same phenomenon is observed in Western countries; as a result, the strategy for solving clinical issues is often expected to arise from a single idea (6). Communitarianism is sometimes preferred to individualism in workplaces and communities (7, 8). The attitudes of healthcare professionals in different specialties toward one another as well as toward other healthcare providers may limit the potential for collaboration and cross-disciplinary work (9). The degree of innovation in epidemiologic studies may be limited when professionals conform to cultural norms and associated values. The presentation of results may be biased toward those that fit within epidemiologists' world views (10). Epidemiologists tend to view issues at the level of large populations and summary statistics such as mean, standard deviation, and proportion.

Japan has relatively low academic job mobility because of the closed nature of the scholarly community and limited opportunities for academics in administrative agencies and private industry. Part of the closed nature of academic employment may be related to hiring based on referral rather than ability. Private companies sometimes hesitate to hire employees with doctoral degrees. Additionally, barriers exist among faculties in Japanese universities. For example, members of the faculties of medicine and of nursing value different academic cultures and qualifications. Because of these attitudes, trends in research publishing and student education can differ among faculties. Occasionally, such attitudes impede collaboration between members of different faculties (e.g., medicine and nursing or medicine and science).

Japanese administrative officers often have lifetime employment and rarely change jobs. Although they may rotate within roles in an organization, very few find academic jobs following administrative work. Hospital clinicians constantly seek scientific evidence to support their clinical practice. However, they are bound by their professional qualifications and clinical duties, and thus rarely publish scientific papers. Many non-academics (those employed outside of universities) want to implement scientific findings into practice (11). However, there are several barriers preventing this in the general population including socioeconomic status and class, obstacles related to healthcare qualifications, and the ability to understand English. Health professionals' views and knowledge are not always shared among the general population (12). Non-health professionals cannot always view study results from a clinical perspective. Epidemiologic research conducted by administrative professionals could identify new social needs and spark widespread interest. This may, in turn, lead to increased diversity in other branches of medical science. Interpretation of study results in the context of different professional perspectives is also important in disseminating results more widely.

In this study, we measured changes during the early and late 2010s in the variety of research topics published in an epidemiologic journal. We aimed to provide a perspective on how epidemiologists from diverse backgrounds can increase innovation and foster cross-discipline interactions.

## Methods

We investigated how the variety of research topics in epidemiology has changed over time. We examined the topics of research articles published in the *Journal of Epidemiology*, the official journal of The Japan Epidemiological Association. The journal accepts submissions from professionals working in the fields of medicine, odontology, pharmacology, nursing, public health administration, nutrition, sports medicine, physical therapy, occupational therapy, the humanities and social sciences, and veterinary medicine.

We assigned all articles published in the journal into categories of epidemiologic subspecialty based on their titles. Articles could be assigned to multiple categories. For example, we categorized a paper entitled “Community social capital and depressive symptoms among older people in Japan: A multilevel longitudinal study” (5) under social epidemiology, psychiatry, and geriatrics. To examine changes in the variety of topics covered in the journal over time, we examined articles published in 2011–2013 and in 2017–2019. We used Fisher's exact test to assess differences in the proportions of articles on each topic comparing 2011–2013 with 2017–2019. The goal of this analysis was to examine changes in epidemiologic research topics over the 2010s. We also calculated the Shannon–Weaver diversity index (H′) (6) to assess changes in topic diversity over time using the following formula:

$$\begin{array}{l} {\text{H}}^{\prime }=-\Sigma \text{Pi log Pi}, \end{array}$$

where Pi, representing the proportion of subset i within the entire set, is sensitive to changes in the relative abundance in a community (13). We also counted the number of categories appearing only in 2011–2013 or in 2017–2019. Using this count, we explored changes in the total number of categories addressed by published articles.

## Results

**Figure 1:**
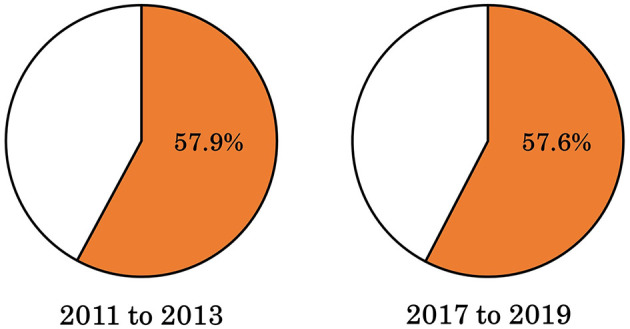
Percentage of papers published in the *Journal of Epidemiology* addressing the top seven research topics (cardiovascular disease, diabetes, cancer, physical activity, nutrition, tobacco, alcohol or internet addiction, and social epidemiology) in 2011–2013 and 2017–2019.

Table 1 summarizes the topics of epidemiologic studies published in the *Journal of Epidemiology* by epidemiologic subspecialty. Overall, the diversity of study topics did not change significantly from 2011–2013 to 2017–2019. The most common topics overall were cancer, cardiovascular disease, diabetes, physical activity and orthopedics, nutrition, tobacco, alcohol and internet addiction, and social epidemiology. Figure 1 shows percentage of papers addressing the top seven research topics (cardiovascular disease, diabetes, cancer, physical activity, nutrition, tobacco, alcohol or internet addiction, and social epidemiology) in 2011–2013 and 2017–2019. Estimates of diversity using the Shannon–Weaver index were similar for the two periods: 4.25 for 2011–2013 and 4.21 for 2017–2019. In 2017–2019, there were greater proportions of studies on perinatal disease, pediatrics, geriatrics, oral/dental disease, disaster medicine, social epidemiology, and disability compared with 2011–2013. The increased focus on children, disaster medicine, periodontal disease, and disability may reflect greater interest in these topics in recent years. The proportions of articles on intractable diseases (rare diseases with unknown etiology requiring long-term treatment) and methodology decreased significantly from 2011–2013 to 2017–2019. Very few topics were observed only in the earlier or the later data set. Only one topic (radiation) appeared in 2017–2019 but not the earlier period, and only one topic (pharmacoepidemiology) appeared in 2011–2013 but not the later period.

**Table 1:** Diversity in topics of articles published in the *Journal of Epidemiology* in 2011–2013 and 2017–2019.

**Epidemiologic topic^†^**	**2011–13**	**Percentage**	**2017–19**	**Percentage**	***P*-value^‡^**
Clinical medicine, kidney disease	4	1.3	8	2.5	0.38
Cancer	27	8.7	32	10.0	0.59
Infectious disease	13	4.2	12	3.7	0.84
Cardiovascular disease	34	10.9	30	9.3	0.51
Digestive disease	3	1.0	6	1.9	0.51
Respiratory disease	8	2.6	4	1.2	0.26
Diabetes	29	9.3	31	9.7	0.89
Perinatal disease	12	3.9	17	5.3	0.45
Pediatrics	11	3.5	19	5.9	0.19
Geriatrics	8	2.6	13	4.0	0.38
Psychiatry	13	4.2	14	4.4	1.00
Intractable disease	7	2.3	1	0.3	0.035
Radiation	0	0.0	1	0.3	1.00
Oral/dental disease	3	1.0	6	1.9	0.51
Nutrition	24	7.7	24	7.5	0.16
Physical activity	27	8.7	25	7.8	0.77
Air pollution	5	1.6	5	1.6	1.00
Occupational medicine	4	1.3	6	1.9	0.75
Disaster medicine	5	1.6	9	2.8	0.42
Social epidemiology, deprivation, income or house	17	5.5	20	6.2	0.74
Genetic epidemiology	13	4.2	4	1.2	0.29
Molecular epidemiology	5	1.6	2	0.6	0.28
Tobacco, alcohol or internet addiction	22	7.1	23	7.2	1.00
Disability and hearing or visual ability	2	0.6	6	1.9	0.29
Methodology	13	4.2	3	0.9	0.011
Pharmacoepidemiology	2	0.6	0	0.0	0.24
Total	311	100	321	100	
Shannon-Weaver's diversity index H′		4.25		4.21	

† *A single study could be categorized under multiple topics*.

‡ *From Fisher's exact test*.

Following our exploratory Shannon–Weiner analysis, we applied Fisher's exact tests of proportion to assess differences in the frequencies of individual topics from 2011–2013 to 2017–2019. We found no significant difference in any topic after adjusting for multiplicity. Overall, topics of epidemiologic studies did not significantly change over the 2010s. This suggests that in Japan, at least, innovation in epidemiologic research may be limited. Therefore, we asked what could be done to bring together diverse professions and translate research results across disciplines.

## Discussion

### Interpretation and Limitations of the Results

The total number of different topics of studies published in the *Journal of Epidemiology* was 311 in 2011–2013 compared with 321 in 2017–2019. In the 2010s, the diversity of topics addressed by published epidemiologic studies did not change significantly. This analysis was based on an overview of trends in study topics in a single journal. We anticipated that the diversity of topics would have changed over time. However, the diversity index and total number of topics addressed by published articles were similar in the two periods, suggesting no significant change.

Our analysis was limited in several ways. First, we assessed the topics of epidemiologic studies published in a single journal. The aim of this analysis was to explore the diversity of topics addressed in epidemiology and to assess how rapidly these topics were changing. Second, we could not evaluate the statistical significance of changes over time in the Shannon–Weaver diversity index. Trends in epidemiologic research depend on the needs of clinical practice and the interests of the general population. Further investigation of academic trends in the publication of epidemiologic research may illuminate social opinions in the medical field. Third, the journal we investigated (the *Journal of Epidemiology*) was the official journal of an academic association, and thus authors were relatively biased toward members of the association. Thus, trends in the diversity of research topics were biased to deflect the interests of Japanese academics and members of The Japan Epidemiological Association.

The results in Table 1 indicate that the topics addressed by epidemiologic studies and the authors' backgrounds cover diverse fields of medical science. The subjects of studies ranged from children to older adults. Papers covered clinical medicine, odontology, nutrition, exercise medicine, and environmental science. Thus, almost all qualified professionals contributed to articles published in the journal.

Most studies covered multiple specialties. This is a typical feature of epidemiologic studies. When publishing the results of an epidemiologic study, researchers need to collaborate with other epidemiologists, clinicians, administrative officers, and laypeople from different backgrounds.

### Research Environments in Japan

Recently in Japan, the profession of epidemiology has become famous because of the ongoing coronavirus disease 2019 (COVID-19) pandemic. Formerly, epidemiologists were almost unknown among the general public. This is potentially because for over 200 years, the Japanese medical community has placed high value on basic science to understand physiological or biochemical causal associations. Conversely, epidemiology, a pragmatic data science based on correlation, was not highly weighed in policymaking or defining clinical guidelines. Recently, epidemiology has achieved enhanced influence in administration, clinical practice, and industry. Although academic, administrative, and clinical employment is highly coveted, the profession of epidemiology needs to be open to everyone to facilitate the use of epidemiologic data.

### Current Roles of Epidemiologists

Epidemiologists in Japan may currently have several roles, whether they are clinicians or informed observers and laypeople. These roles include translation of epidemiologic results, analyzing data to help drive changes in clinical practice, improving medication plans, and developing new techniques for clinical epidemiologic studies. The diversity of these roles means that studies supported by epidemiologists can sometimes achieve innovative results (14).

It has become commonplace for scientific journals to request that authors ask an epidemiologist or biostatistician to assess the statistical methods of submitted manuscripts (15). Advising on statistical methods is an important role of epidemiologists. Biostatisticians can check manuscripts from a mathematical perspective, but epidemiologists can also check the statistical appropriateness of the study and statistical design. Therefore, epidemiologists fit between clinicians and biostatisticians and must understand both clinical needs and mathematics. Careers in epidemiology, therefore, should be open to professionals from different backgrounds, including clinicians and biostatisticians.

### Conveying Epidemiologic Study Results to the General Population

It is common for clinicians and epidemiologists to work together as a team to design clinical epidemiologic studies, collect and analyze data, and report findings. These findings, expressed in non-technical terms, may appear in mainstream newspapers and magazines, as well as in media aimed at clinicians. Specialist media may provide detailed information on exposures, outcomes, and interpretations in the context of previous studies. Newspapers tend to provide more general information that is not particularly useful in clinical practice. This means that epidemiologists need to seek a balance between value for clinicians and the public.

In presenting epidemiologic work, illustrations may help to make data more understandable. Tables and figures should be designed so that they are comprehensible to both researchers in similar fields and to other professionals working in diverse areas. We believe that findings should be understandable by high school-level students (16) and be openly accessible online (17) to reach the broadest possible audience. The word “publish,” after all, means to make findings available publicly.

### Interpreting Research Results to Reflect Different Values and Cultures

To make their findings clear to individuals with different cultures and values, epidemiologists need to learn how to convey technical concepts to non-academics. In Japan, there is rarely a need to communicate with people who hold different cultural values in the workplace because there has been limited social mobility. However, this has begun to change because of the end of career-long employment in an era when intellectual labor and creative thinking are required rather than factory work with industry-specific experience. Companies that produce information (e.g., Google, Amazon, Facebook, and Apple) have become dominant in place of those producing machines (e.g., Toyota or Honda). Instead of skilled labor, creativity and idea-driven thinking that understands the needs of public health and the general population is now needed for life-long employment. This means that epidemiologists may need to work with colleagues with different values and even change workplace several times over their lives. For example, epidemiologists may learn about public health values in administrative agencies and about commercial values in companies. Administrative and commercial values are often related to clinical values because both administrative agencies and commercial companies often focus on helping people remain healthy (6). In general, scholars work toward ideals, public administrators aim to provide social benefit within a given budget, and businesspeople strive for commercial gain and social contribution. However, the aims of the work conducted by these three groups often overlap; all of them pursue the public interest. The differences between them may lie primarily in terminology.

Epidemiologists may have difficulty in creating illustrations that are understandable to non-academics. For example, when presenting an odds ratio of two for mortality among cigarette smokers, journalists may understand this to mean that smoking doubles the mortality rate. This is not entirely correct in a mathematical sense (18) but may be considered good enough for interpretation by non-academics. Another example is the use of hazard ratios with adjustment of covariates. Here, academics might maintain that the hazard ratio needs to be interpreted using assumptions of proportional hazard (19) and appropriate covariate selection. However, it may be acceptable for non-academic purposes to ignore these assumptions.

Researchers must select the most appropriate strategies for conveying research findings to those in other professions. The optimal approach differs by specialty. For example, in reporting the impact of seasonal influenza infection on hospitalization within a region, local clinicians may use the number of hospitalizations (20). However, government officials may find it more informative to see the monthly hospitalization rate displayed as a graph, and it may therefore be necessary to provide both forms of information.

### Exchanging Ideas on Clinical Issues and Data Analysis

Unlike clinicians, epidemiologists do not necessarily have intimate knowledge of patients' needs. Similarly, clinicians may not know how to analyze data to drive changing practice. It can therefore be helpful for clinicians and epidemiologists to collaborate to explore a particular issue. This may also help clinical epidemiology to survive as a career in the era of big data.

In Japan, there may be barriers between clinicians and epidemiologists in the conduct of clinical epidemiologic studies. These barriers could include different values and cultures arising from departmental priorities, duty hours, primary needs, career course, and criteria for performance evaluation.

From 2015 to 2017, we undertook a case-control study to investigate the risk of immune thrombocytopenic purpura risk associated with simultaneous vaccinations (21). This was a successful example of collaboration between clinicians, administrative officers, and epidemiologists. We sparked useful discussions spanning clinical interests, administrative needs, and the validity of epidemiological methods. These clinical interests, administrative needs and methodological priorities sometimes conflicted. However, after several discussions, all parties agreed. Finally, the results were reported to the Ministry of Health, Labor and Welfare and subsequently published. We consider it essential for individuals of different professional backgrounds in a study group to discuss and exchange their needs and cultural values.

### Lessons From the COVID-19 Pandemic

Historically, collaborations among politicians, government administrators, administrative officers, clinicians, epidemiologists, data scientists, economists, commercial companies, and citizens has been required to control the spread of pandemics. Collaborations were simplified because the issue was evident and urgent to all parties; technology aided their communication and assisted the development of countermeasures. The COVID-19 pandemic may teach us valuable lessons in how to ask assistance from other professionals at remote sites as well as in how to efficiently share information. Although different professional cultures can sometimes prevent mutual understanding, new scientific innovations might be generated by the admixture of values from different cultures (1, 2).

### Collaborations Among Individuals From Diverse Backgrounds

Combining different academic specialties can produce new disciplines (1). For example, cross-disciplinary work between medicine and evolutionary biology created the field of evolutionary medicine (22), which has provided new interpretations of why humans develop disease. Cross-disciplinary research on circadian rhythms and psychiatry produced the field of sleep science. The field of psychology has also provided “nudge” interventions to help prevent disease (23). Throughout history, academic fields have intersected, converged, and diffused. Collaboration has been at the departmental, institutional, or national level and has been aided by development of technologies and communication tools.

Collaboration can be achieved by bringing together people from different backgrounds. However, the experience of different roles and organizations can also encourage individuals to produce new ideas themselves. Collaboration between individuals may be made difficult by physical distance and psychological issues. However, friction of ideas within an individual can also lead to innovation. In solving a complex issue, bringing together different ideas within a single brain can often be extremely powerful, including in the field of epidemiology.

There is very low job mobility in Japan (24). Working for a single company was considered ideal in the period of high economic growth after the Second World War. However, Japan has now entered an era in which many people change jobs several times during their lives. In the future, epidemiologists will have much more diverse backgrounds and skills, which may be crucial in exploring new ideas and innovations in the field.

## Conclusion

Our analysis of changes in article titles published in an epidemiologic journal suggested little change in the diversity of research topics in the 2010s. However, topics over this decade covered all clinical qualifications and specialties. Thus, in epidemiologic studies, communication with individuals with diverse backgrounds is essential. The intersection between clinicians, administrators and epidemiologists has the potential to foster scientific innovation. This could be important both in situations in which each role is taken by a different person and in which an individual has worked in several fields and brings these distinct experiences together. Research results can be most easily tailored to an audience by researchers who are also part of that audience. Scientific progress has been a challenge for researchers for many years. Changing attitudes by encouraging movement between workplaces and bringing together professionals from different backgrounds has the potential to create new and innovative results, catalyze innovation, and drive scientific progress.

## Data Availability Statement

The original contributions generated for the study are included in the article/supplementary material, further inquiries can be directed to the corresponding author/s.

## Author Contributions

HY: original draft preparation, conceptualization, methodology, analysis, investigation and review, and editing. MM: original draft preparation, investigation and review, results interpretation, and editing. ZY: resources, results interpretation, and editing. All authors contributed to the article and approved the submitted version.

## Conflict of Interest

The authors declare that the research was conducted in the absence of any commercial or financial relationships that could be construed as a potential conflict of interest.
